# Is attendant at delivery associated with the use of interventions to prevent postpartum hemorrhage at home births? The case of Bangladesh

**DOI:** 10.1186/1471-2393-14-24

**Published:** 2014-01-16

**Authors:** Ndola Prata, Suzanne Bell, Martine Holston, Mohammad A Quaiyum

**Affiliations:** 1 Bixby Center for Population, Health and Sustainability, School of Public Health, University of California at Berkeley, 229 University Hall, UC-Berkeley, Berkeley, CA 94720-7360, USA; 2 Bixby Center for Population, Health and Sustainability, School of Public Health, University of California at Berkeley, 17 University Hall, UC-Berkeley, Berkeley, CA 94720-7360, USA; 3 Venture Strategies Innovations, 2401 East Katella Avenue, Suite 400, Anaheim, California 92806, USA; 4 icddr, b, Centre for Reproductive Health, GPO Box 128, Dhaka 1000, Bangladesh

**Keywords:** Bangladesh, Maternal mortality, Postpartum hemorrhage, Misoprostol, Delivery mat, Home delivery, Traditional birth attendant

## Abstract

**Background:**

Hemorrhage is the leading cause of maternal mortality in Bangladesh, the majority of which is due to postpartum hemorrhage (PPH), blood loss of 500 mL or more. Many deaths due to PPH occur at home where approximately 77% of births take place. This paper aims to determine whether the attendant at home delivery (i.e. traditional birth attendant (TBA) trained on PPH interventions, TBA not trained on interventions, or lay attendant) is associated with the use of interventions to prevent PPH at home births.

**Methods:**

Data come from operations research to determine the safety, feasibility, and acceptability of scaling-up community-based provision of misoprostol and an absorbent delivery mat in rural Bangladesh. Analyses were done using data from antenatal care (ANC) cards of women who delivered at home without a skilled attendant (N = 66,489). Multivariate logistic regression was used to assess the likelihood of using the interventions.

**Results:**

Overall, 67% of women who delivered at home without a skilled provider used misoprostol and the delivery mat (the interventions). Women who delivered at home and had a trained TBA present had 2.72 (95% confidence interval, 2.15-3.43) times the odds of using the interventions compared to those who had a lay person present. With each additional ANC visit (maximum of 4) a woman attended, the odds of using the interventions increased 2.76 times (95% confidence interval, 2.71-2.81). Other sociodemographic variables positively associated with use of the interventions were age, secondary or higher education, and having had a previous birth.

**Conclusion:**

Findings indicate that trained TBAs can have a significant impact on utilization of interventions to prevent PPH in home births. ANC visits can be an important point of contact for knowledge transfer and message reinforcement about PPH prevention.

## Background

It is estimated that postpartum hemorrhage (PPH) accounts for 25% of global maternal death [1,2]. In low-resource settings, the vast majority of women deliver at home, unattended by a skilled provider and with inadequate resources should emergency obstetric care be needed [1,3]. Contributing to the difficulty of addressing the problem is the low predictive probability of PPH [4] and the speed at which PPH kills; 88% of women are dead within 4 hours of delivery [3]. The compounding effect of the biological and contextual circumstances helps to explain why PPH continues to be the leading cause of maternal death in low-resource settings despite concerted efforts to address the problem.

In Bangladesh, the maternal mortality ratio is 194 per 100,000 live births [5]. Hemorrhage is the leading cause of maternal mortality in Bangladesh, accounting for 31% of total maternal deaths; postpartum hemorrhage (PPH) in particular is largely responsible for these deaths [5]. Many deaths due to PPH occur at home where approximately 77% of births take place [5]. Of the 2.4 million births that take place at home annually, only 4.3% use a medically trained provider, a negligible change from 3.5% in 2001 [5]. Reducing the number of deaths attributable to PPH will require community-based use of evidence-based technologies that can be used by low-level or lay birth attendants [6].

Ideally a skilled provider would administer active management of the third stage of labor (AMTSL) immediately following delivery, an intervention that can prevent more than half of PPH cases [7]. With such a high proportion of home births, nearly all of which have no skilled attendant and no clear trend away from such practices, it is unrealistic to rely exclusively on AMTSL administration by a skilled provider as a means of preventing PPH. Bringing the most effective component of AMTSL, the uterotonic drug [4], to the community-level has the potential to prevent PPH attributable death among the most vulnerable women.

Misoprostol is an affordable, thermostable uterotonic that has been demonstrated to be safe and effective in the prevention of PPH at the community-level [8-10]. Prophylactic use of misoprostol at home births in comparison to a placebo can reduce the risk of PPH by 24% to 47% [8,9]. Unlike oxytocin, the preferred uterotonic in the management of PPH, it can be administered orally/sublingually instead of parenteral. This allows low-level providers and even the delivering woman herself to administer misoprostol; operations research has demonstrated the feasibility, safety, effectiveness, and acceptability of this approach [11-17]. Based on a review of existing evidence, the WHO added misoprostol to the Essential Medicines List for the prevention of PPH in 2011 [18] and in its most recent guidelines for PPH prevention and treatment, the WHO recommends the use of misoprostol by community and lay healthcare workers where skilled attendance and oxytocin are not available [19].

Even with prophylactic use of misoprostol at home deliveries, some women will experience PPH and need immediate transfer to a facility for further treatment. Identifying PPH cases presents its own difficulties. When using visual observation alone, amount of blood loss is often underestimated, which can result in delayed referral and subsequent fatality [20]. Using a measurement tool, like the “kanga” in Tanzania, can allow whoever is present at a home delivery to more accurately gauge blood loss and help ensure timely transfer when necessary [21]. Use of misoprostol in conjunction with a blood measurement tool provides a means of preventing the leading cause of maternal mortality at the community-level, helping to address the inequity of healthcare access in low-resource settings.

This paper aims to determine whether the attendant at delivery (i.e. traditional birth attendant (TBA) trained on PPH interventions, TBA not trained on interventions, or lay attendant, which includes a family member or neighbor) is associated with the use of interventions to prevent PPH at home births.

## Methods

### Intervention

Researchers conducted operations research to determine the safety, feasibility, and acceptability of scaling-up community-based provision of misoprostol and an absorbent delivery mat at home births in rural Bangladesh. The study was implemented in the six northern-most rural districts of Rangpur Division, Bangladesh. From July 2009 to January 2011, study personnel enrolled a total of 118,594 women, 77,337 of which delivered during the data collection period. All women who enrolled were registered antenatal care (ANC) clients of Rangpur Dinajpur Rural Services (RDRS), Bangladesh, a local non-governmental organization (NGO) that has been providing services to the rural poor in the study area since 1972.

RDRS has 62 community health workers (CHWs) and nearly 600 TBAs working in their reproductive health program, which is based out of 203 ANC clinics throughout the six study districts. TBAs are responsible for recruiting and registering women in RDRS’ ANC program by going door-to-door in their respective communities. They also attend ANC at their affiliated clinic once a week and attend home deliveries of RDRS registered women who request their services. CHWs lead the clinic-based ANC and provide oversight of the TBAs who work in their upazilas (sub-districts). Prior to the start of the study, RDRS was providing clean delivery kits (CDKs) to women at a nominal cost. CDKs contained a sterile razor blade, thread, cotton gauze, a plastic sheet on which to deliver, and a bar of soap. TBAs attending home deliveries used only visual estimation to determine PPH cases and were instructed to immediately transfer all suspected cases to a nearby facility.

For the operations research, 600 μg of misoprostol (three 200 μg tablets) and an absorbent delivery mat for measurement of blood loss (developed by Dr. MA Quaiyum and colleagues at the International Centre for Diarrhoeal Disease Research, Bangladesh (icddr,b)) were incorporated into the existing CDK [21]. RDRS did not charge for these additional CDK components. CHWs continued to provide the CDKs to women through ANC at 32 weeks or beyond, and TBAs began distributing CDKs at the time of delivery if women did not receive the CDK through ANC. TBAs and CHWs received two days of training on the new additions to the CDK. The training included information on myriad aspects of misoprostol use (function, dosage, timing of administration, side effects and their management, etc.) and the absorbent delivery mat. The training also covered information regarding the identification of high-risk pregnancies, danger signs of pregnancy, referral procedures, stages of labor, newborn resuscitation, maternal infection, and general use of the CDKs. Trainers particularly focused on PPH and other pregnancy complications that require referral. Evidence from previous analyses indicate that TBAs learned and retained key information about the new technologies and utilized the tools correctly and to the satisfaction of patients [22].

An information, education, and communication (IEC) campaign accompanied the operations research. Women, family members, and communities learned about the new CDK through extensive IEC efforts outside of service delivery. Women received additional information about the new CDK components from CHWs at clinic-based ANC.

In this analysis, we used program data to determine if, among those who delivered at home without a skilled provider, the use of the study interventions (misoprostol and the absorbent delivery mat) is associated with the attendant at delivery.

### Analysis

Data analyzed are from ANC cards. All women enrolled in the study received an ANC card that was filled out by CHWs during ANC (N = 118,594) and then during a post-delivery checkup for those who delivered during the study period (N = 77,337). It contains information on basic demographics, index pregnancy, obstetric history (if applicable), ANC information, enrollment status, whether or not she received the CDK, and delivery information (i.e. place of delivery, outcome, CDK use, attendant, referral, etc.). All data collected on this card were entered into a database using Epi Info™ version 3.5.1 [23] and analyzed using STATA® version 11.0 [24].

All analyses were done with the subpopulation of women who delivered at home during the study period (N = 67,611) and who did not have a doctor or nurse present (N = 66,489). Univariate and bivariate (Chi-square) analyses were conducted. Using results from the bivariate analyses, all covariates associated with the use of the study interventions (dependent variable) at the p ≤ 0.20 level were included in the multivariate model. Logistic regression with odds ratios and 95% confidence intervals was then used to determine whether attendant at delivery had a significant impact on the likelihood of use of the interventions. Observations with missing values for any of the variables included in the logistic regression were dropped. This resulted in a 4% reduction in the number of women included in the final analysis.

Ethical approval for the operations research was provided by the Committee for the Protection of Human Subjects at the University of California, Berkeley (protocol #2008-8-24) and by icddr,b’s research administration in Bangladesh. The RDRS staff did not employ any coercive methods to collect information from participating women and women were free to not answer any questions without penalty or impact on service delivery. The data collected were de-identified and this secondary data analysis did not require additional ethical approval or authorization.

## Results

Overall, 67% of women who delivered at home without a skilled provider used misoprostol and the absorbent delivery mat (the interventions) to prevent PPH (Table 1). Across most age categories, 60-70% of women used the interventions, but only 50% of women ages 45–49 used the interventions (Table 1). Similar proportions of women used the interventions regardless of level of education and gravidity (60-70%). Use of the interventions was positively correlated with number of ANC visits, with women who attended only one ANC visit being least likely to use the interventions (20%) and women who attended four ANC visits being most likely (84%) (Table 1). Percent of women who used the interventions was highest among those who had an RDRS trained TBA present (71%) compared to those who had a non-RDRS TBA (48%) or lay person (54%) present (Table 1). Bivariate results revealed that all covariates, which include attendant at delivery, age, education, gravidity, and number of ANC visits, were significantly associated with the use of study interventions at the p ≤ 0.20 level (data not shown).

**Table 1:** Use of misoprostol and absorbent delivery mat among women who delivered at home without a skilled provider by sociodemographic and pregnancy characteristics (N = 66,489)*

	**% that used interventions**	**N**
Total	67.4	64,413
Age		
15-19	67.8	20,558
20-24	66.9	21,759
25-29	67.9	15,209
30-34	67.1	5,008
35-39	62.9	1,487
40-44	66.2	151
45-49	50.0	40
Education		
None	63.0	12,380
Primary	64.4	19,104
Secondary	70.8	29,474
College	69.3	3,066
Gravidity		
1	66.9	25,430
2+	67.6	38,909
Number of ANC visits		
Average		3.2
1	19.7	6,844
2	42.4	8,922
3	66.5	15,196
4	84.2	33,419
Attendant at delivery		
Lay person	53.8	355
TBA	47.7	10,738
RDRS trained TBA	71.4	53,291

*N categories do not add up to 66,489 due to missing responses from some women.

Based on the bivariate results, all covariates were included in the multivariate model. Women who delivered at home and had an RDRS trained TBA present had 2.72 times the odds of using the interventions compared to those who had a lay person present (95% confidence interval, 2.15-3.43) (Table 2). Compared to women ages 15–19, women ages 20–24 and 25–29 were *not* more likely to use the interventions, nor were the eldest women surveyed (ages 45–49). However, women ages 30–34, 35–39, and 40–44 were increasingly more likely to use the interventions, and these findings were statistically significant (odds ratio 1.15, 1.16, and 1.64, respectively). Reaching secondary school or college was similarly significantly associated with increased odds of using the interventions (odds ratio 1.43 and 1.41, respectively), as was having had a previous pregnancy (odds ratio 1.16) (Table 2). Each additional ANC visit a woman attended was associated with 2.76 times the odds of using the interventions (95% confidence interval, 2.71-2.81).

**Table 2:** Logistic regression investigating whether attendant at home birth is associated with use of interventions to prevent PPH (N = 63,702)

	**OR**	**p-value**	**95% CI**
Attendant at delivery			
Lay person	--	--	Reference
TBA	1.02	0.896	0.80, 1.29
RDRS trained TBA	2.72	<0.001	2.15, 3.43
Age			
15-19	--	--	Reference
20-24	0.96	0.184	0.90, 1.02
25-29	1.06	0.125	0.98, 1.14
30-34	1.15	0.004	1.05, 1.26
35-39	1.16	0.041	1.01, 1.33
40-44	1.64	0.014	1.11, 2.43
45-49	1.14	0.719	0.56, 2.34
Education			
None	--	--	Reference
Primary	1.00	0.943	0.94, 1.05
Secondary	1.43	<0.001	1.36, 1.51
College	1.41	<0.001	1.27, 1.55
Gravidity			
1	--	--	Reference
2+	1.16	<0.001	1.09, 1.22
Number of ANC visits	2.76	<0.001	2.71, 2.81

## Discussion

Our primary hypothesis, that having an RDRS trained TBA present at the delivery would be associated with increased use of the interventions, was correct. To our knowledge, this is the first study to examine if type of delivery attendant is associated with the use of community-based interventions to prevent PPH at home births. Even though all women who received the CDK also received messages on how to use the interventions, it seems that having an attendant specifically trained to administer the interventions significantly increased utilization. Women who had an RDRS trained TBA present, in comparison to a lay person, were nearly three times more likely to use misoprostol and the delivery mat; women with a regular TBA present were not more likely to use them. A strong explanation for this association is that the RDRS trained TBA prompted the use of the interventions during the delivery. This explanation would justify the training of TBAs on these evidence-based technologies to increase utilization in other low-resource settings where home births are common and will continue to be so for many years. The strong association between number of ANC visits and use of the interventions shows the potential impact that could come from the increased interaction and message reinforcement by healthcare providers via ANC. In each appointment, the importance of these interventions and the risk of pregnancy were reiterated. This could have led women to be more knowledgeable and more motivated to use misoprostol and the delivery mat, and repeatedly hearing the instructions could have made them more confident that they would use the tools correctly.

Findings related to other covariates demonstrate interesting associations. Increased age was not linearly associated with increased use of the interventions, but it did trend in that direction. This finding could be interpreted in conjunction with the fact that having had a previous pregnancy was associated with increased use of the interventions. Younger women who have never been pregnant and who may not know as much about the dangers and potential complications of pregnancy may have been less inclined to utilize these interventions in comparison to older women, who may have experienced complications first hand in previous pregnancies, or know other women who have. An alternative explanation is that these older women (ages 30–44) might have more autonomy and feel more empowered to make choices about their healthcare utilization and delivery practices than younger, nulliparous women, who may be taking direction from their mothers-in-law. This potential demonstration of autonomy is likely why women with secondary and college education are also more likely to use these interventions in comparison to women with no formal schooling. The eldest women’s (ages 45–49) lesser utilization of the interventions (although not statistically significant) could be a result of having survived several pregnancies by that age and not being interested in incorporating new, unfamiliar technologies into their home delivery practices.

A limitation of this study is that all data were self-reported by the women. Women provided information on all the covariates during their first antenatal care appointment with an RDRS CHW, and information on attendant at delivery and use of the interventions was later self-reported at a postnatal appointment. Self-reported data can be less accurate and be subject to social desirability bias. Direct observation was not possible given the number of women enrolled in the project, but efforts were made to collect pill packets from all women after delivery to assess whether the misoprostol had been used or not. Despite these efforts, if women falsely reported use of the interventions (and we do not believe women who did use the interventions would have any incentive to falsely report non-use), this would result in a differential misclassification that would bias our results toward the null. Thus our results would be a conservative estimate of the association between attendant at delivery and use of PPH prevention interventions. An additional limitation is that the data are cross-sectional, thus we cannot make claims of causality and alternative explanations for the findings could be made. Women who have an RDRS trained TBA present at delivery or who attend more ANC could be systematically different from mothers who had a regular TBA or lay person present, or who attended fewer ANC visits. Some unmeasured characteristic (like motivation or knowledge) could have caused the women to be more likely to have an RDRS trained TBA present, attend more ANC appointments, *and* to use the interventions. Alternatively, both explanations could be true and they could reinforce each other.

Further evidence is needed to determine the direction of the relationship between attendant at delivery/number of ANC visits and use of interventions. As access to misoprostol in low-resource settings increases, governments and non-profits will want to work to increase knowledge and utilization of this important technology that can reduce PPH at home births by as much as 50% [9]. If training a community-based cohort of TBAs on misoprostol (and a blood measurement tool) and strengthening recruitment of women for ANC will increase utilization of these technologies, such efforts could in turn reduce the impact of maternal morbidity and potentially mortality attributable to PPH in the parts of the world that are most burdened by this complication. Existing evidence from this project demonstrates that TBA training on the use of these interventions can be highly effective [22]. The two interventions were safely and correctly used at home births by the TBAs and data regarding TBA practices and knowledge indicate adherence to protocol and high knowledge retention even 18 months after the training [22]. Despite these positive training results, it is important to always consider the limitations and risks of utilizing lay healthcare workers to administer medical interventions. Trainings should emphasize that providers only use misoprostol immediately following delivery (using the recommended dosage after ensuring there is no twin) and that misoprostol is never to be used for any other purpose.

Side effects, although not addressed in this study, are an important consideration that program planners and policymakers should take into account. Health interventions, especially those implemented at home, should always aim to have as few side effects as possible and the potential benefits of the intervention should be weighed against the risk of side effects. In the case of misoprostol for PPH prevention, the potential reduction in the risk of PPH is greater than the potential harm of the transient side effects associated with 600 μg of oral misoprostol use; none of the side effects have been associated with long term morbidity or mortality. Studies have demonstrated that lower doses of misoprostol are associated with similar levels of uterine pressure but less adverse effects, which seems to indicate that lower doses could be used with similar levels of efficacy [25]. A non-inferiority trial will be required to determine whether 400 μg or 200 μg of misoprostol is as effective as 600 μg in the prevention of PPH.

Additionally, the quest to conclusively demonstrate whether prophylactic use of misoprostol reduces mortality continues. A recent review of existing evidence found that misoprostol use for the prevention of PPH neither increased nor decreased the risk of mortality or severe morbidity. It is important to note that none of the studies included in this review were individually powered to detect significant changes in the risk of mortality, but the findings nonetheless question whether similar projects to the one presented here would actually reduce maternal mortality due to PPH [26].

Making evidence-based interventions available is the first step to improving the safety of home deliveries in low-resource settings, but the availability of these interventions alone will not ensure usage. In areas where complete coverage of SBAs is far from a reality, training existing TBAs to use these tools can be an effective way to increase their utilization among women who have these TBAs present during home delivery, as findings from this study indicate.

## Conclusion

Given misoprostol’s potential to reduce PPH, it is increasingly being made available in low-resource settings where the majority of PPH occurs. Findings indicate that a trained TBA’s presence at delivery can potentially have a significant impact on utilization of interventions to prevent PPH. PPH prevention efforts targeting home births that use advanced distribution of misoprostol should thus consider targeting delivery attendants for intervention-specific training. Programs that aim to increase the safety of home deliveries should also perhaps increase efforts to enroll women in ANC and encourage attendance at multiple appointments, as ANC attendance was found to be associated with increased utilization of misoprostol and the absorbent delivery mat. These efforts, in the context of a reliable continuum of care, may help to reduce the morbidity and mortality associated with PPH in Bangladesh and other low-resource countries.

## Competing interests

The authors declare that they have no competing interests.

## Authors’ contributions

NP devised the larger operations research study design and the research question for this study. She also contributed to the writing and editing of the manuscript. SB conducted the statistical analyses and was integral to the writing of the manuscript. MH was involved in the management of the operations research, the cleaning and coding of the data, and the editing of the manuscript. MQ was a co-Principal Investigator in the larger operations research, oversaw data collection, and helped with the editing of this manuscript. All authors read and approved the final manuscript.

## Pre-publication history

The pre-publication history for this paper can be accessed here:

http://www.biomedcentral.com/1471-2393/14/24/prepub
